# “Safety and utility of percutaneous liver biopsy in hematopoietic stem cell transplant pediatric recipients: a retrospective study”

**DOI:** 10.1186/s12885-016-2603-8

**Published:** 2016-08-02

**Authors:** Natalia Maximova, Massimo Gregori, Francesca Barbieri, Antonio Pizzol, Aurelio Sonzogni

**Affiliations:** 1 Bone Marrow Transplant Unit, Institute for Maternal and Child Health - IRCCS Burlo Garofolo, via dell’Istria 65/1, 34137 Trieste, Italy; 2 Department of Radiology, Institute for Maternal and Child Health - IRCCS Burlo Garofolo, Trieste, Italy; 3 Department of Pediatrics, Institute for Maternal and Child Health - IRCCS Burlo Garofolo, University of Trieste, Trieste, Italy; 4 Department of Pediatrics, University of Turin, Turin, Italy; 5 Department of Pathology, Ospedale Beato Papa Giovanni XXIII, Bergamo, Italy

**Keywords:** Allogeneic hematopoietic stem cell transplantation, Liver graft versus host disease, Percutaneous liver biopsy, Pediatric patients, Child emotional distress

## Abstract

**Background:**

Liver biopsies in pediatric hematopoietic stem cell transplantation (HSCT) patients are as and effective when performed at bedside in the Bone Marrow Transplant Unit (BMTU) than in the Day Surgery Unit (DSU), with better patient compliance and lower emotional distress for these children.

**Methods:**

The study group consisted of 45 children who underwent allogeneic HSCT. We reviewed 68 liver biopsies performed between April 2006 and September 2015. 12 (17.6 %) biopsies were performed in the DSU and 56 (82.3 %) in the BMTU; nine (13.2 %) prior to HSCT and 59 (86.7 %) after HSCT. Pre-procedural behavioral status (subjective score) was evaluated by pediatric transplant physicians by filling in a questionnaire employing a three-point scale: “calm and cooperative”, “agitated and non-cooperative” or “frightened and suffering”. Objective score was obtained measuring patient’s heart rate before the procedure and comparing it with mean heart rate.

**Results:**

Patients who underwent the procedure at the BMTU experienced less emotional distress than those who underwent it in the DSU: 58.3 % of patients treated at the DSU were agitated as compared with 16.1 % of those treated at the BMTU (*p* < 0.01). Among the 59 biopsies performed after HSCT, 41 (69.5 %) were taken from symptomatic patients for a diagnostic purpose and 18 (30.5 %) in asymptomatic ones in order to rule out hepatic GVHD. Among these 18 procedures, GVHD was diagnosed in 16 (88.9 %) cases. Minor complications occurred in about 17 % of procedures (12 biopsies), at a rate of 25 % for the DSU location compared with 16 % for the BMTU location. Only two major complications were reported, one in the DSU and one in the BMTU.

**Conclusion:**

Liver biopsy performed at bedside in HSCT patients does not carry a higher risk of adverse events than the same procedure performed in the DSU and has lower emotional distress associated with better patient compliance, thus contributing significantly to a higher standard of care.

## Background

Over recent years, allogeneic hematopoietic stem cell transplantation (HSCT) has become an important technique for treating pediatric diseases, especially hematological and oncological disorders and congenital errors, including congenital immunodeficiency. Great improvements in survival rates have been achieved owing to better control of these diseases, introduction of less aggressive conditioning regimens, better management of graft versus host disease and infectious complications, and improvements in human leukocyte antigen typing, which has made highly accurate selection of unrelated donors possible. Thus, the benefits of HSCT now outweigh the associated risks.

However, despite the decrease in transplant-related mortality, previously unknown and difficult to treat complications are common and can result in suboptimal management. One of the most significant of these complications is liver dysfunction, which has been classified by Forbes et al. by cause as follows: graft-versus-host disease (GVHD), drug hepatotoxicity, viral hepatitis, sepsis, veno-occlusive disease, and disease recurrence. A significant proportion of liver complications are still classified as of undetermined cause [1].

Liver biopsy is currently the gold standard means of identifying the nature of hepatic lesions. This invasive procedure is commonly considered high risk for pediatric recipients of allogeneic HSCT because of the possibility of fatal complications such as massive bleeding, gastric perforation, respiratory distress during anesthesia, sepsis, hemothorax, and ascites caused by hepatic failure [2, 3].

We herein report a small retrospective series of 45 pediatric patients with allogeneic hematopoietic stem cell transplants who had undergone ultrasound-guided liver biopsy (single or multiple) between April 2006 and September 2015. Our hypotheses were that it is safer and more effective to perform liver biopsies at the bedsides of such patients than in the Day Surgery Unit (DSU) and that it is associated with better patient compliance and less emotional stress for the child, thus contributing significantly to a higher standard of care.

## Methods

### Patients

Medical records of all pediatric patients with allogeneic hematopoietic stem cell transplants who had undergone percutaneous ultrasound-guided liver biopsy between April 2006 and September 2015 in the Bone Marrow Transplant Unit (BMTU) or DSU of the Institute for Maternal and Child Health, Institute for Scientific Research, Burlo Garofolo, in Trieste, Italy were retrospectively reviewed and analyzed. The Institutional Review Board approved the study protocol (reference no. 1105/2015). Written informed consent for biopsies had been obtained from the parents of all subjects.

During the observation period, 68 percutaneous ultrasound-guided liver biopsies were performed. From April 2006 to April 2012, 12 biopsies (17.64 %) were performed in the DSU, which is external to the BMTU; these biopsies involved a duty radiologist and the entire operating room team. From April 2012 to September 2015, 56 biopsies (82.5 %) were performed on 36 patients in a laminar air flow room in the BMTU; these biopsies involved a pediatric team that included pediatricians certified in Pediatric Advanced Life Support (PALS) and BMTU nurses certified in Pediatric Basic Life Support.

The study group consisted of 45 children with allogeneic hematopoietic stem cell transplants. They were divided into the following four age groups: infants, under 1 year; young children, from 1 to 5 years; children, from 6 to 10 years; and adolescents, from 10 to 18 years [4].

The following data were collected for each study patient: sex, body mass index, primary disease, conditioning regimen, type of donor, GVHD prophylaxis, number of HSCTs, pre-transplant hepatopathy, magnetic resonance imaging (MRI) quantification of hepatic iron stores [5], liver function tests, and platelet count at the time of liver biopsy. Baseline patient characteristics are listed in Table 1.

**Table 1:** Baselines characteristics of the sample of the study

Baseline characteristics		*n*°	%	DSU (%)	BMTU (%)
Patient *n*°		45	100	7 (15.5)	38 (84.4)
Gender	F	15	33.3	5 (16.3)	13 (86.7)
	M	30	66.6	5 (16.7)	25 (83.3)
Age	<1 years	2	15.5	15.5 1 (50.0)	1 (50.0)
	1–5 years	15	24.4	2 (13.3)	13 (86.7)
	6–10 years	13	28.8	1 (7.8)	12 (92.3)
	10–18 years	15	31.1	3 (20.0)	12 (80.0)
Primary disease	AL	27	60.0	4 (14.8)	23 (85.2)
	Hemoglobinopathy	1	2.2	-	1 (100)
	Inherited disease	6	13.3	1 (16.7)	5 (83.3)
	MDS	10	22.2	2 (20.0)	8 (80.0)
	Solid tumor	1	2.2	-	1 (100)
Number of HSCT	1	37	82.1	6 (16.3)	31 (83.7)
	2	5	11.1	1 (20.0)	4 (80.0)
	3	2	4.4	-	2 (100)
Type of Donor	Haploidentical	6	13.3	4 (66.7)	2 (33.3)
	Matched related	18	40.0	2 (11.1)	16 (88.9)
	Matched unrelated	21	86.6	1 (4.8)	20 (95.2)
Conditioning regimen	Myeloablative	39	86.6	7 (17.9)	32 (82.1)
	RIC	6	13.3	-	6 (100)
Gilbert syndrome	Yes	9	20.0	1 (11.1)	8 (88.9)
	No	36	80.0	6 (16.6)	30 (83.3)
Pre transplant hepatopathy	No	18	40.0	1 (5.5)	17 (94.4)
	Acquired metabolic disorders	1	2.2	-	1 (100)
	Drug Hepatotoxicity	1	2.2	-	1 (100)
	Inherited metabolic disease	2	4.4	1 (50.0)	1 (50.0)
	Iron overload	21	46.6	4 (19.0)	17 (80.9)
	Viral hepatitis	2	4.4	1 (50.0)	1 (50.0)
MRI quantification LIC	Patients undergoing MRI	42	93.3	4 (9.5)	38 (90.5)
	Normal liver	4	9.5	-	4 (100)
	Slight overload	16	38.0	-	16 (100)
	Moderate overload	8	19.0	2 (25.0)	6 (75.0)
	Major overload	14	33.3	2 (14.3)	12 (85.7)
Obesity (BMI >30)		6	13.3	2 (33.3)	4 (66.7)

*DSU* Day Surgery Unit, *BMTU* Bone Marrow Transplant Unit, *F* Female, *M* Male, *AL* Acute leukemia, *MDS* Myelodisplasic syndromes, *RIC* Reduced intensity conditioning, *MRI* Magnetic resonance imaging, *LIC* Liver iron concentration, *BMI* Body mass index

Twenty-seven children (60.0 %) had acute leukemia, ten myelodysplastic syndromes (22.2 %), six inherited diseases (13.3 %), one a solid tumor (2.2 %), and one hemoglobinopathy (2.2 %).

All medical records of biopsy procedures and the post-biopsy observation period, including any treatment for any complications, were examined. Additionally, changes in management prompted by biopsy results were extracted.

### Pretransplant conditioning

Thirty-nine of the 45 patients had undergone myeloablative conditioning and the remaining six non-myeloablative conditioning regimens. Recipients of haploidentical or matched unrelated donors had also received antithymocyte globulin.

GVHD prophylaxis was performed with calcineurin inhibitor associated to mycophenolate mofetil and prednisone in the matched unrelated and haploidentical donor group.

### Percutaneous ultrasound-guided liver biopsy

The indications for biopsy were persistent or progressive alteration noted at 2 weekly follow-up assessments of at least one clinical or laboratory marker of liver impairment or cholestasis, abnormal radiological findings, or one or more lesions suggestive of hepatic parenchymal infection. Contraindications to biopsy were as follows: severe ascites, refractory thrombocytopenia, failure to correct coagulation defects with transfusions of plasma or specific coagulation factors, and severe respiratory or hemodynamic impairment.

**Fig. 1:**
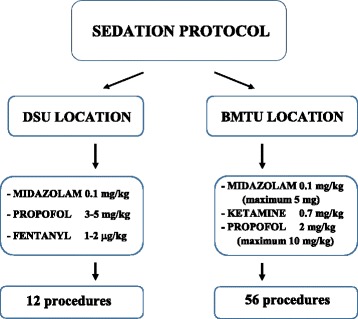
Sedation protocols categorized by location

All 45 patients had undergone percutaneous biopsies under deep sedation (five or six Ramsay scale sedation scores [6]). Sedation in the SDU was induced by midazolam, propofol and fentanyl administration, while in the BMTU by midazolam, ketamine and propofol. Both sedation protocols are illustrated in Fig. 1. In the operating room, induction and maintenance of anesthesia were performed by anesthesiologists, in the BMTU by a PALS-certified pediatrician. Additionally, all patients had received prophylactic treatment with parenteral antifibrinolytic agent tranexamic acid during the 30 min before the procedure. From April 2012 onwards, sedation was administered by a resident pediatrician at the BMTU who had received 4 weeks of training in the operating room and practiced sedation for a further month, during which tutoring was given by a pediatric anesthesiologist [7] and by the radiologist who had performed all the biopsy procedures; this radiologist had extensive experience in pediatric interventional radiology.

All procedures were supported by sonography performed by the interventional radiologist who performed the biopsy. A mapping sonogram was used to identify the best approach for hepatic biopsy (generally in the 10th intercostal space in the anterior axillary line) and to ensure a safe needle pathway that minimized the risk of accidental puncture of interposing bowel, gall bladder, lung, right kidney, or intrahepatic vessels and ducts. A MyLab™30 US machine (Esaote, Genova, Italy) with a multiband frequency probe (2.5–6.6 MHz) without a biopsy adaptor was used in all cases.

Needle size was determined by age and body mass index. An Ester automatic core biopsy device (Biopsybell SRL, Mirandola, Italy) with a 16- or 18- gauge 150-mm needle with a drawing area of 20 mm was employed. Twenty liver biopsies (29.4 %) were obtained with 16-gauge, 46 (67.6 %) with 18-gauge, and two (2.9 %) with 20-gauge needles.

Immediately after the biopsy, a further sonography was performed to check for possible bleeding or other complications. In the absence of complications, all patients were observed for 8 h after liver biopsy in our Transplant Unit, in which central monitoring is available. Sonography was usually performed the next day on inpatients and the evening of the same day on outpatients. Patients with acute hemorrhage were examined promptly by sonography CT scan with contrast medium.

Complications were defined as adverse events that occurred as a direct result of the procedure. These were classified as minor (local bleeding evaluable by sonography, local or radiating pain) or major: bleeding requiring transfusion or intervention (Hb decrease > 2 g/dL in the 8 h after the procedure), infection, perforation, unintentional organ injury, postoperative complications directly related to the administration of anesthesia, and specific organ complications such as cholangitis and pneumothorax [8].

Pre-procedural behavior status was evaluated by the pediatric transplant physician of our BMTU, so that the same person made the assessments during liver biopsy in both the DSU and BMTU. To give this subjective score, some minutes before every procedure the pediatrician evaluates the patient’s behavior and asks each patient a few questions to assess anxiety on a 3-point scale: “calm and cooperative”, “agitated and non-cooperative” or “frightened or suffering”. Adverse effects of ketamine administration were reported as “frightened or suffering”. Objective score was obtained measuring patient’s heart rate immediately before administration of anesthetic drugs and comparing it with mean heart rate. The mean heart rate for each of four age groups was calculated using the heart rates documented during entire admission. Heart rate data was obtained by pulse oximetry measurements in both locations (DSU and BMTU). Tachycardia were defined as a 20 % increase above the mean heart rate [9].

### Statistical analysis

Statistical analysis was performed with IBM SPSS statistical software v.20. Data are presented as means ± standard deviation. Comparisons between categorical variables were evaluated with Fisher’s exact test. Comparison between numerically limited variables were evaluated with Wilcoxon test. A *p*-value of <0.05 was considered statistically significant.

## Results

The clinical and biological characteristics of the 45 patients categorized by their location are presented in Table 1. The median time between HSCT and the biopsy procedure was 156 days ± 141 (range 29–641 days) for subjects who had biopsies taken after receiving their transplants; additionally, biopsies were taken before the HSCT in nine (20 %) cases. Of the 68 liver biopsies, 12 (17.6 %) were performed in the DSU and 56 (82.3 %) in the BMTU; 31 (45.6 %) were performed on inpatients and 37 (54.4 %) on outpatients. Suitable samples were obtained in 98.5 % of procedures and microscopic examination of biopsy material revealed abnormalities in 66/68 samples (97.0 %).

In general, platelet counts of more than 100 × 10^9^/L and international normalized ratio (INR) values lower than two prior to the procedure are preferred. Pre-procedure platelet counts were >140 × 10^9^/L in all patients, platelet transfusions having been administered prior to the procedure in 29 cases. None of the HSCT patients in the study had an INR greater than two (median INR 1.07 ± 0.16); three patients received fresh frozen plasma transfusions before the procedure to reduce their high risk of bleeding. The patients’ pre-procedural liver function test results (mean ± standard deviation) were: total bilirubin 0.79 ± 0.69 mg/dL; direct bilirubin 0.37 ± 0.41 mg/dL; alanine transferase 59.79 ± 56.47 U/L; and alkaline phosphatase (ALP) 179.54 ± 100.4 U/L. Ascites was observed in 5/of 68 procedures (7.35 %). Pre-transplant hepatopathy had been present in 27 patients (60.0 %), a high proportion being associated with iron overload (46.6 %).

Nine (13.2 %) liver biopsies were performed prior to the HSCT: four to define iron overload disease, one to stage chronic hepatitis B disease, one to confirm the healing of hepatitis C disease after treatment with sofosbuvir and ribavirin, and two to diagnose hepatopathy of uncertain origin.

Of the 59 liver biopsies (86.76 %) performed after HSCT, 41 (69.49 %) were performed on symptomatic patients for diagnostic purposes and 18 (30.50 %) on asymptomatic patients to determine whether hepatic GVHD was present; 16 of these 18 patients (88.9 %) were found to have GVHD.

In 36 liver biopsies (52.9 %), the pathological findings led to changes in clinical management, either because a new diagnosis was made or resolution or progression of GVHD identified. The finding of resolution of GVHD led to suspension of immunosuppressive treatment, whereas confirmation of GVHD or evidence of progression of this condition prompted intensification of therapy. In 32 hepatic samples (47.1 %) there were no histological changes since the previous diagnosis, and these patients’ therapy was therefore not modified (Table 2).

**Table 2:** Histological diagnosis and clinical management

Clinical characteristics		N° (%)	DSU (%)	BMTU (%)
Total biopsy		68 (100)	12 (17.6)	56 (82.3)
Number of biopsy per patient	1	17 (37,7)	3 (17.6)	14 (82.3)
	2	17 (37,7)	3 (17.6)	14 (82.3)
	≥3	11 (24,4)	1 (9.1)	10 (90.9)
Histological diagnosis	Drug Hepatotoxicity	2 (3,9)	1 (50.0)	1 (50.0)
	AIH	1 (1,4)	-	1 (100)
	GVHD	24 (35,2)	4 (16,7)	20 (83.3)
	GVHD + IO	19 (27,9)	3	16
	GVHD + IO + Infection	1 (1,4)	-	1 (100)
	GVHD + IO + MD	2 (2,9)	-	2 (100)
	Infection	4 (5,8)	1 (25.0)	3 (75.0)
	IO + Drug toxicity	4 (5,8)	1 (25.0)	3 (75.0)
	IO + Infection	1 (1,4)	1 (100)	-
	IO + MD	1 (1,4)	-	1 (100)
	Iron overload	6 (8,8)	1 (16.7)	5 (83.3)
	Metabolic Disorders	2 (2,9)	0	2 (100)
	Normal	1 (1,4)	0	1 (100)
Changes in clinical management	No	32 (47,1)	3 (9.4)	29 (90.6)
	Yes	36 (52,9)	9 (25.0)	27 (75.0)
Outcome	Improved	33 (48,5)	6 (18.2)	27 (81.8)
	Recovered	13 (19,1)	2 (15.4)	11 (84.6)
	Not changed	19 (27,9)	2 (10.5)	17 (89.5)
	Worsened	1 (1,4)	1 (100)	0
	Death	2 (2,9)	1 (50.0)	1 (50.0)

Histological diagnosis and clinical management. *DSU* Day surgery unit, *BMTU* Bone marrow transplant unit, *AIH* autoimmune hepatitis, *GVHD* Graft versus host disease, *IO* Iron overload, *MD* Metabolic disease

The rate of biopsy procedures without complication was 75 % in the DSU group and 83.9 % in the BMTU group, and there was no significant difference between the two groups. Minor complications, namely local bleeding and pain at the site of the needle insertion, occurred in about 17 % of procedures (12 biopsies): 3 patients (24.9 %) from the DSU group versus 9 patients (16 %) from the BMTU group. Two major complications were reported: one patient from DSU group had hemorrhage requiring transfusion and one patient from the BMTU had cholangitis requiring antibiotic treatment. Moreover, medication, namely a single dose of an opioid analgesic, was needed in only 9 % of all procedures (six biopsies), with no significant difference between the two groups (Table 3).

**Table 3:** Complications of biopsies performed in the DSU versus BMTU and behavioral status of patients: Evaluation of major and minor complications after biopsy procedure and treatment adopted for complications; Pre-procedure behavioral status of the patient is also reported

Complications and patient’s behavioral status		DSU location	BMTU location
No Complication		9 (75.0)	47 (83.9)
Minor complications	Local bleeding	2 (16.6)	4 (7.1)
	Local pain	1 (8.3)	5 (8.9)
Major Complications	Bleeding requiring transfusion	1 (8.3)	-
	Infection	-	1 (1,8)
Complication treatment	None	9 (75.0)	47 (83.9)
	Morphine	2 (16.6)	4 (7.1)
	Surgical procedure or transfusion	1 (8.3)	-
	Tramadol	-	5 (8.9)
Pre-procedure status	Calm and cooperative	5 (41.7)	47 (84.0)
	Agitated and non-cooperative	6 (50.0)	8 (14.3)
	Frightened or suffering	1 (8.3)	1 (1.8)

As to behavioral status, patients were reported to be “calm and cooperative” in approximately 80 % of pre-procedure evaluations. This difference in incidence of being calm and cooperative between the two procedure locations was statistically significant (*p* < 0.01). Additionally, 58 % of patients who had undergone biopsies in the DSU were assessed as agitated or non-cooperative versus 16 % of those in the BMTU (also significant; *p* < 0.01) (Table 3). The number of tachycardia events (objective score) was signicantly higher in the DSU than in the BMTU (58.3 % versus 16.1 %; *P* < 0.01). Unfortunately the sample is very heterogenous, and many of the age groups, especially in the DSU population, are to small for statistical comparison. The pre-procedural heart rates and the incidence of tachycardia events are reported in the Table 4.

**Table 4:** Baseline heart rate and tachycardia events categorized by location: BMTU, Bone Marrow Transplant Unit; DSU, Day Surgery Unit

Age groups	BMTU LOCATION				DSU LOCATION			
	N° of biopsies (%)	Baseline heart rate	Tachycardia (%)	*P* ^a^ Value	N° of biopsies(%)	Baseline heart rate	Tachycardia (%)	*P* ^a^ Value
<1 year	2 (2.9)	136.5 (±7.78)	-	-	-	-	-	-
1 - 5 years	8 (11.7)	121.1 (±11.87)	1 (12.5)	NS	3 (4.4)	124.6 (±4.72)	1 (33.3)	NS
6 – 10 years	17 (25.0)	101.47 (±13.48)	6 (35.3)	<0.05	5 (7.3)	107 (±8.12)	4 (80.0)	<0.05
>10 years	29 (42.6)	78.2 (±8.94)	2 (6.9)	<0.05	4 (5.9)	75 (±5.88)	2 (50.0)	0.067^b^
Total	56 (100)	-	9 (16.1)	<0.01	12 (100)	-	7 (58.3)	<0.01

^a^Wilcoxon test

^b^borderline value not quite significant (the *t*-test for paired data yielded a value of p <0.05, but it not suitable in comparison of small cases numbers)

*BMTU* Bone marrow transplant unit, *DSU* Day surgery unit

## Discussion

Several small studies have suggested that liver biopsies should be considered as risky in pediatric patients with cancer or hematological disease [10] and that the morbidity and mortality of this procedure is higher in children than in adults [11, 12]. However, hepatopathy, which has a morbidity rate of 80 % and mortality rate close to 14 % in HSCT recipients, is a common complication in these patients [3]. Clinical and laboratory findings are non-specific in many of these cases and insufficient to make a definitive diagnosis.

Over the 3 years from April 2012 to September 2015, 57 liver biopsies were performed on 36 patients with allogeneic hematopoietic stem cell transplants in our institution, which represents a more than 10-fold increase in the biopsy rate. The main reason for this dramatic jump is the increasing necessity of making histological-based diagnoses because of the availability of targeted therapies and the need to choose the optimal management strategy. One consequence of increasing our liver biopsy rate was that after April 2012, the only patient who developed Grade 3–4 hepatic GVHD had developed Grade 4 GVHD after receiving a donor lymphocyte infusion for cryptosporidiosis.

MRI quantification of liver iron concentration had not yet been introduced at the time our first two asymptomatic patients underwent liver biopsy to evaluate iron overload. These two children’s main laboratory abnormalities were in ferritin and ALP concentrations. Examination of their biopsies confirmed hepatic iron overload and showed a mild chronic GVHD. Because of the latter unexpected finding, all subsequent patients with at least one indicator of cholestasis (abnormally high concentrations of ALP and/or bile acids) underwent liver biopsy to determine whether subclinical GVHD was present. This policy resulted in histological confirmation of GVHD in 16 of 18 asymptomatic patients (88.89 %), the management of all of whom was modified accordingly. None of these patients showed any subsequent evidence of GVHD.

There were only a few complications in our series. Local bleeding and pain at the site of the needle insertion occurred in about 17 % of procedures (12 biopsies). There were only two major complications (2.9 %): a biliary tract infection and a hemothorax. The first biopsy had been performed in the DSU and the second in the BMTU. Both patients were in poor general condition, the former having Grade 4 GVHD and the latter having undergone three transplants.

Our results confirm the usefulness of liver biopsy and highlight that, in terms of complications, the procedure is as safe as other procedures routinely performed on such patients, such as lumbar puncture and bone marrow aspiration.

The change in location of the procedure from the DSU to the BMTU has not altered its safety. On the contrary, the rate of minor complications decreased from 25 to 16 % after this change, which is likely not a clinically significant difference considering how few patients had undergone the procedure in the DSU.

To date, little attention has been paid to the management of the emotional stress that can be experienced by young patients when they undergo invasive procedures such as liver biopsies. All of the patients who are referred to the BMTU have undergone many diagnostic and therapeutic procedures, some of which have been invasive (such as bone marrow aspiration and lumbar puncture). During their long stays in the BMTU, these children develop close and trusting relationships with the inpatient team. Our findings demonstrate that when procedures are performed in the child’s room by a dedicated pediatrician, an intervention radiologist, and members of the day-to-day inpatient team, the patient is less afraid or agitated [13]. Moreover, use of midazolam for sedation reduces post-procedural anxiety because this drug has been shown to enhance anterograde amnesia in pediatric patients [14, 15].

When we analyzed the reports of behavioral status, we noticed that 84 % of the BMTU population were reported as being calm and collaborative prior to the procedure. Moreover, when we compared the behavioral status and the tachycardia events of patients treated in the DSU with those at the BMTU, we observed higher emotional distress with a higher incidence of tachycardia in those treated at the DSU, more of whom were assessed as agitated and non-cooperative. Indeed, 58.3 % of patients who underwent biopsies in the DSU were agitated or non-cooperative versus 16.1 % of those in the BMTU. Thus, our patients who underwent the procedure in their own rooms experienced less emotional distress than those who underwent it in the less familiar and friendly environment of the DSU, an important consideration for children.

## Conclusions

In this small study, we found that liver biopsy is safe in pediatric patients who have undergone HSCT, few complications having been encountered. Moreover, our study shows that biopsies performed in the patient’s room can have the same outcomes as those performed in the DSU but are associated with better patient compliance and less emotional stress for the child, thus contributing significantly to a higher standard of care.

## Abbreviations

ALP, alkaline phosphatase; BMI, body mass index; BMTU, Bone Marrow Transplant Unit; DSU, Day Surgery Unit; GRE, gradient-recalled-echo; GVHD, graft-versus-host disease; HCST, hematopoietic stem cell transplantation; INR, international normalized ratio; MRI, magnetic resonance imaging; PALS, Pediatric Advanced Life Support.
